# Neutralizing Antibody Response to the AreXvy Respiratory Syncytial Virus Vaccine in Lung Transplant Recipients: Assessment Against Reference and Seasonal Strains

**DOI:** 10.3390/vaccines13040398

**Published:** 2025-04-11

**Authors:** Liran Levy, Dafna Yahav, Mark Benzimra, Yael Bezalel, Tomer Hoffman, Neta Shirin, Tomer Sinai, Menucha Jurkowicz, Ofir Deri, Noa Matalon, Milton Saute, Yaniv Lustig, Eyal Nachum, Michael Peled, Ital Nemet, Michal Mandelboim

**Affiliations:** 1 Institute of Pulmonary Medicine, Sheba Medical Center, Ramat Gan 5262000, Israel; mark.benzimra@sheba.health.gov.il (M.B.); yael.bezalel@sheba.health.gov.il (Y.B.); noa.matalon@sheba.health.gov.il (N.M.); michael.peled@sheba.health.gov.il (M.P.); 2 Sheba Lung Transplant Program, Sheba Medical Center, Ramat Gan 5262000, Israel; dafna.yahav@sheba.health.gov.il (D.Y.); tomerhof@gmail.com (T.H.); milton.saute@sheba.health.gov.il (M.S.); 3 Faculty of Medical & Health Sciences, Tel Aviv University, Ramat Aviv, Tel Aviv 6997801, Israel; neta.shirin@sheba.health.gov.il; 4 Infectious Diseases Unit, Sheba Medical Center, Ramat Gan 5262000, Israel; 5 Central Virology Laboratory, Public Health Services, Ministry of Health, Sheba Medical Center, Tel Hashomer, Ramat Gan 5262000, Israel; tomer.sinai@sheba.health.gov.il (T.S.); menucha.jurkowicz@sheba.health.gov.il (M.J.); yaniv.lustig@sheba.health.gov.il (Y.L.); ital.nemet@sheba.health.gov.il (I.N.); michal.mandelboim@sheba.health.gov.il (M.M.); 6 Department of Epidemiology and Preventive Medicine, School of Public Health, Faculty of Medical and Health Sciences, Tel Aviv University, Tel Aviv 6997801, Israel; 7 Department of Cardiac Surgery, Leviev Cardiothoracic and Vascular Center, Sheba Medical Center, Ramat Gan 5266202, Israel; eyal.nachum@sheba.health.gov.il

**Keywords:** respiratory syncytial virus (RSV), Arexvy, vaccine, lung transplant, immunogenicity

## Abstract

Background: Respiratory Syncytial Virus (RSV) is a significant cause of morbidity and mortality among lung transplant (LTx) recipients. Therapeutic options are limited, emphasizing the importance of prevention. The Arexvy^®^ vaccine (RSVPreF3) showed promising efficacy among immunocompetent adults; however, data on its immunogenicity in solid organ transplant recipients remain unclear. Methods: A single-center retrospective cohort study, including all LTx recipients who were vaccinated with Arexvy in February 2024. Baseline and follow-up serum samples (1, 3, and 6 months post-vaccination) were analyzed for antibody responses using a commercial RSV ELISA kit and micro-neutralization assays against historical reference RSV A/B ATCC strains and seasonal RSV strains. Adverse events were documented. Results: A total of 28 recipients received the vaccine. Twenty-one (75%) were male, and the median age was 62 years (interquartile range [IQR], 53–67). The median time from transplant was 486 days (IQR, 243–966). Vaccination elicited strong immunogenic responses, demonstrating a twofold increase in ELISA-determined antibody levels at one month post-vaccination, which were sustained for six months. At one month, 67% of recipients had antibody levels exceeding the cutoff threshold. Micro-neutralization assays showed a significant increase in neutralizing antibodies against all tested variants (RSV A/B ATCC and seasonal RSV A/B), with titers remaining at least twofold higher than pre-vaccination levels. No serious adverse events were observed. Conclusions: Our findings demonstrate a sustained antibody response to the Arexvy^®^ vaccine in a cohort of LTx recipients, with antibody titers sustained over six months. Further research is needed to assess the long-term durability of the immune response and the potential immunogenicity of this vaccine in LTx populations.

## 1. Introduction

Respiratory Syncytial Virus (RSV) is a significant cause of lower respiratory tract infection (LRTI) in both the pediatric population [1,2] and older adults, particularly those aged over 60 years, where it contributes significantly to morbidity and mortality [3,4,5]. Within the context of lung transplantation, recipients are especially vulnerable to infections due to their immunocompromised status, making RSV a leading cause of respiratory tract infections in these individuals. Furthermore, RSV infections have been linked to poor allograft outcomes [6,7,8,9,10]. Despite the serious implications of RSV infections, the risk factors associated with LRTI and mortality within the solid organ transplant (SOT) population remain inadequately characterized. Known risk factors include young children under two years of age, recent transplantation, lung or multivisceral transplantation, and recent episodes of rejection [11]. Currently, there is a lack of strong evidence regarding the best therapeutic approaches for managing RSV infections in lung transplant (LTx) recipients [12,13,14]. Recent guidelines offer a weak recommendation for therapy with ribavirin, with or without intravenous immunoglobulin (IVIG) or corticosteroids, due to the limited evidence derived from observational studies [15]. Given the absence of a specific antiviral treatment for RSV, the primary management strategy focuses on supportive care, which aims to alleviate symptoms and provide necessary respiratory support. Ultimately, the best approach is the prevention of RSV infection, highlighting the importance of vaccination in this vulnerable population.

The Arexvy^®^ vaccine (GlaxoSmithKline Biologicals, Durham, NC, USA), a recombinant AS01E adjuvanted vaccine based on the RSV fusion glycoprotein F (RSVPreF3), has been initially approved by the U.S. Food and Drug Administration (FDA) for preventing RSV-related LRTI in adults aged 60 years and older [16]. Despite the promising efficacy data from clinical trials demonstrating Arexvy’s ability to prevent RSV-related LRTI [17], the effectiveness of this vaccine in SOT recipients remains inadequately characterized. The unique immunosuppressive therapies employed in these patients may hinder the vaccine’s effectiveness, as evidenced by previous studies indicating that SOT recipients often exhibit diminished immune responses to various vaccinations [18]. This study aimed to assess the immunogenicity of the Arexvy vaccine against reference and seasonal RSV strains in LTx recipients and to evaluate the safety of the vaccine in this population.

## 2. Methods

### 2.1. Study Design, Population and Follow-Up Protocol

This single-center, retrospective cohort study utilized prospectively collected data and was approved by the Institutional Research Ethics Board (Approval #1590-24-SMC). As data collection was performed as part of routine clinical care and all analyses were conducted on anonymized samples, the need for individual informed consent was waived. The study population included all adult LTx recipients followed at the Sheba Medical Center LTx clinic. Due to budget constraints limiting vaccine availability, the first 28 eligible LTx recipients were vaccinated in February 2024, at the end of the RSV season in Israel, and subsequently included in the study. As vaccination was not administered at a uniform time point post-transplant, recipients were at varying intervals after transplantation when they received the vaccine. Exclusions were applied only for contraindications to the vaccine, specifically for individuals with a history of severe allergic reactions (e.g., anaphylaxis) to any component of the Arexvy^®^ vaccine. Patients were routinely followed every 3–6 months as part of standard clinical care, with instructions to contact the clinic for any respiratory symptoms or fever between visits. Adverse events were monitored, including injection-site reactions (swelling, erythema, pain), systemic reactions (fever, arthralgia, myalgia, headache, fatigue), and any other events reported within 7 days after the receipt of the vaccine. Clinical and laboratory data, including blood samples, were collected at baseline (pre-vaccination) and at 1, 3, and 6 months post-vaccination (within a ±2-week window), and retrospectively extracted from clinical records for analysis.

### 2.2. Clinical Care of Lung Transplant Recipients

#### 2.2.1. Immunosuppression Protocol

Maintenance immunosuppression: Induction immunosuppression was not routinely used. Standard immunotherapy consisted of tacrolimus (target levels: 10–15 ng/mL), mycophenolic acid 1000–2000 mg/day and methylprednisone 0.5 mg/kg for three days, followed by prednisone 0.5 mg/kg/d, gradually tapered to 0.25 mg/kg/d over three months and later generally tapered to 0.15 mg/kg/d at 6 months and 0.075 mg/kg/d at 12 months post-transplant.

Acute cellular rejection (ACR): Episodes of ACR were categorized utilizing the grading criteria outlined in the International Society for Heart and Lung Transplantation (ISHLT) guidelines [19]. Grade A1 episodes without clinical symptoms or significant changes in spirometry were not treated. Grade A1 episodes associated with clinical symptoms or grade A2 or higher were typically treated unless contraindicated by a concurrent infectious process. In addition to the optimization of maintenance immunosuppression, the first-line treatment of ACR consisted of intravenous methylprednisolone 10 mg/kg daily for 3 days, followed by a prednisone taper starting at 0.5 mg/kg/day and decreasing by 5 mg every five days back to the baseline dose. Patients with repeat ACR (accompanied by clinical symptoms, a drop in lung function, or grade A2 or higher) usually received repeat steroid bolus or a second-line treatment such as anti-thymocyte globulin (ATG).

#### 2.2.2. Antimicrobial Prophylaxis

All patients received prophylaxis against Pneumocystis jirovecii Pneumonia with Trimethoprim-sulfamethoxazole (800 + 160 mg thrice weekly) and 6 months of valganciclovir prophylaxis (900 mg daily, adjusted for renal function). Patients seronegative for CMV prior to transplant who received an organ from a CMV seropositive donor (D+/R−) were given 12 months of prophylaxis. Antifungal prophylaxis was not universally employed; instead, targeted antifungal therapy was initiated for patients deemed to be at greater risk for invasive fungal disease based on surveillance bronchoscopy results.

### 2.3. Microbiological Materials and Methods

#### 2.3.1. Viral Isolation of RSV Variants

RSV A (ATCC VR-26, 1956) and RSV B (ATCC VR-955, 1977) lineages, along with seasonal RSV A (R17535/Israel) and B (R15474/Israel) strains, were propagated in vitro and used to evaluate antibody response. Human kidney cells served as the host cells and were seeded in 24-well plates. The cells were then incubated with the RSV variants for three days. Following incubation, the supernatants were collected, and the attached cells were detached via mechanical scraping. To ensure the complete release of virions, the harvested cells underwent two freeze–thaw cycles. The cell debris was discarded, and the supernatant was combined with the initially collected supernatant.

An aliquot of 500 µL from the combined supernatants was transferred to 75 cm^2^ flasks containing confluent human kidney cells. The flasks were gently agitated at 37 °C for two hours before adding 20 mL of 2% FCS MEM-EAGLE medium. Following four days of incubation, the supernatants were collected, and the attached cells were processed as previously described to ensure the release of any remaining virions. The supernatants were then aliquoted and stored at −80 °C as viral stock.

#### 2.3.2. ELISA

RSV IgG antibodies in sera were determined by quantitative ELISA (SERION ELISA classic, Institute Virion/Serion GmbH, Würzburg, Germany), following the manufacturer’s instructions. To compensate for normal test variations, a reference standard serum sample was used in each individual test run. The quality control certificate values were applied for the calculation of the IgG concertation in every sample, and results were quantified as “U/mL”. The evaluation table included in each test kit designates samples with anti-RSV IgG ≥ 15 U/mL as immunized to RSV (positive), between 10 and 15 U/mL as borderline immunized, and below 10 U/mL as unimmunized to RSV (negative).

#### 2.3.3. RSV Micro-Neutralization Assay

The neutralization potential of inactivated sera from LTx recipients was evaluated against RSV A (ATCC), RSV B (ATCC), and the seasonal RSV A and B strains. VERO-ATCC cells, at a concentration of 20 × 10^3^ cells/well, were seeded in sterile 96-well plates containing 10% FCS MEM-EAGLE medium and incubated at 37 °C for 24 h. Subsequently, 100 TCID50 of each RSV variant was incubated with inactivated and serially diluted patient sera (from 1:8 to 1:16,384) for 60 min at 37 °C. The virus–serum mixtures were then added to the VERO-ATCC cells. The plates were incubated at 37 °C for ten days, after which the monolayers were fixed and stained with 1% Gentian Violet. The neutralizing capacity of each serum sample was determined by identifying the highest serum dilution at which no cytopathic effect was observed. Serum dilutions of 1:10 or higher were considered neutralizing.

#### 2.3.4. Sample Preparation for Sequencing

Viral RNA was extracted from RSV ATCC strains, as well as seasonal RSV A and B strains obtained from hospitalized patients who tested positive for RSV during the winter of 2023–2024. The fusion protein gene was amplified using the OneStep RT-PCR Kit (QIAGEN, Hilden, Germany) with gene-specific primers. To clean up unincorporated primers and dNTPs, EppicFast (A&A Biotechnology, Gdansk, Poland) was used on the cDNA. Sequencing was performed using the Big Dye^®^ Terminator v1.1 Cycle Sequencing Kit (ABI Prism^®^, Foster City, CA, USA) following the manufacturer’s protocol. The resulting cDNA was purified with the BigDye^®^ Xterminator Purification Kit (ABI, Foster City, CA, USA). Samples were processed and sequenced using the Genetic Analyzer 3500 (ABI/HITACHI, Poway, CA, USA).

#### 2.3.5. Phylogenetic Analysis

Genetic variations between the ATCC reference strains and the contemporary circulating strains (i.e., seasonal strains) were evaluated by sequencing the fusion protein gene of both ATCC strains and from 17 RSV-positive clinical samples collected from hospitalized patients during the winter of 2023–2024. For phylogenetic analysis, the nucleotide sequences of the samples were compared using Sequencher^®^ 5.0 software (Gencodes Corporation, Ann Arbor, MI, USA) [20].

### 2.4. Statistical Analysis

Descriptive statistics summarized the baseline demographic and clinical characteristics of the study cohort, including the median and interquartile ranges (IQR) for continuous variables and frequencies with percentages for categorical variables. Plots of log-transformed neutralizing antibodies and geometric mean titers (GMTs) with a 95% confidence interval were obtained using GraphPad Prism 5.0 (GraphPad Software, Inc., San Diego, CA, USA). Differences in antibody levels prior to and 6 months post vaccination were assessed via Wilcoxon signed rank tests. Chi-square analysis was used to assess the differences in percentage positivity of RSV cases prior to and 6 months post vaccination. All statistical tests were two-tailed, with a significance level of *p* < 0.05. Analyses were performed using GraphPad Prism 5.0 (GraphPad Software, Inc., San Diego, CA, USA).

## 3. Results

### 3.1. Baseline Characteristics of Lung Transplant Recipients

The baseline clinical characteristics of the 28 LTx recipients included in the study are summarized in Table 1. The median age at the time of transplantation was 59 years (IQR 50–65), and at the time of vaccination, 62 years (IQR 53–67). The median time from transplant to vaccination was 486 days (IQR 243–966). Among the participants, 21 (75%) were male and 25 (89.3%) had undergone bilateral lung transplantation. Pulmonary fibrosis was the most common underlying lung disease, affecting 19 patients (67.9%), followed by chronic obstructive pulmonary disease in 4 patients (14.3%) and cystic fibrosis in 2 patients (7.1%). Most participants (71.4%) were CMV serostatus donor-positive and recipient-positive (D+R+). Mild, localized side effects including swelling, erythema, and pain were the only adverse events, reported by eight (28.6%) participants. Notably, no RSV infections were documented in this population before or after vaccination during the study period.

### 3.2. Seropositivity and Antibody Activity (ELISA Results)

**Figure 1:**
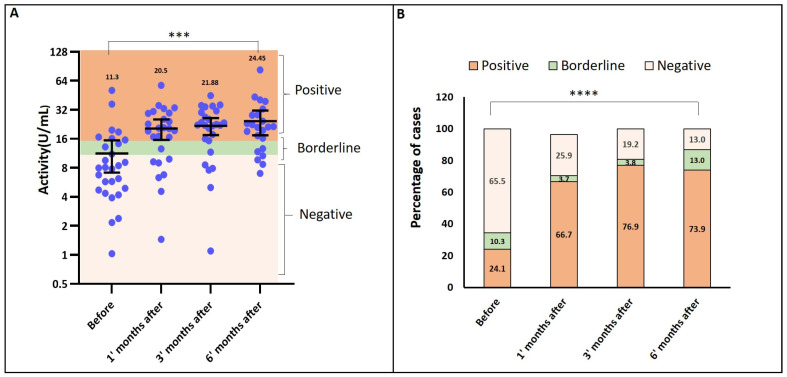
Binding of patients’ antibodies to commercial RSV antigen A. (**A**) Percentage distribution of IgG activity results: The percentage distribution of IgG activity levels, as measured by the RSV ELISA kit, was analyzed across the same four time points, providing insights into the antibody response dynamics over time. Statistical significance is denoted as *** *p* = 0.00035. (**B**) IgG activity levels over time: IgG activity (U/mL) was assessed at four time points (pre-vaccination, and at 1, 3, and 6 months post-vaccination) using a commercial RSV ELISA kit. Optical density (OD) values obtained from the assay directly corresponded to the RSV-binding antibody titers in each sample. Statistical significance is denoted as **** *p* < 0.0001.

Prior to vaccination, 25% of participants were seropositive for RSV-specific antibodies, as measured by ELISA. One month after vaccination, seropositivity increased to 67%, compared to 25% at baseline. By six months post-vaccination, 74% of participants maintained antibody levels above the predefined threshold of 10 U/mL. The mean RSV antibody activity increased significantly, from 11.3 U/mL at baseline to 24.45 U/mL at six months post-vaccination (*p* < 0.0001) (Figure 1A). The percentage distribution of ELISA results demonstrated a significant rise in RSV antibodies at all post-vaccination time points *p* = 0.00035 (Figure 1B).

### 3.3. Neutralizing Antibody Response (Micro-Neutralization Assay)

**Figure 2:**
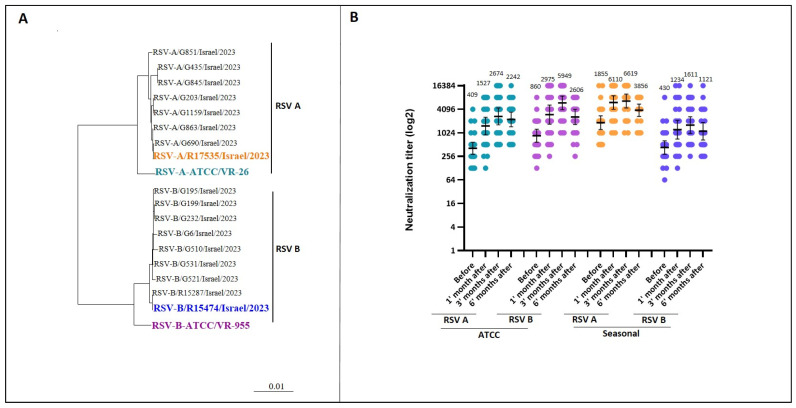
Neutralizing antibody response before and after vaccination. (**A**) Phylogenetic tree: A Bayesian maximum clade credibility time-scaled phylogenetic tree was constructed using BEAST, based on the RSV fusion protein gene sequences from RSV A and B ATCC reference strains and 17 RSV-positive samples collected from hospitalized patients during the winter of 2023–2024. (**B**) Neutralizing Antibody Titers Over Time: Sera collected at four time points (before vaccination and at 1, 3, and 6 months post-vaccination) were tested for neutralizing antibodies against RSV A ATCC (light blue), RSV B ATCC (purple), seasonal RSV A (orange), and seasonal RSV B (blue) using a micro-neutralization assay. The geometric mean titers (GMT) are indicated alongside 95% confidence intervals, represented by error bars.

We evaluated RSV vaccination efficacy against historical reference strains (RSV A VR-26 and RSV B VR-955 ATCC), resembling the vaccine strain (RSV A VR-1540 ATCC), and against circulating strains from winter 2023–2024. Genetic variations were assessed by sequencing the RSV fusion protein from the ATCC strains mentioned above and 17 RSV-positive samples. The phylogenetic analysis revealed substantial genetic differences between historical reference strains and currently circulating RSV strains, showing the evolving nature of the virus (Figure 2A).

Immune responses were assessed at baseline and 1, 3, and 6 months post-vaccination using micro-neutralization assays. Neutralization experiments utilized the cultured and sequenced VR-26 and VR-955 strains, and seasonal strains (RSV A R17535 and RSV B R15474) (Figure 2B). The micro-neutralization assay demonstrated a significant increase in neutralizing antibodies across all four RSV variants by six months post-vaccination. For RSV A ATCC, the geometric mean titer (GMT) rose from 409 to 2242 (*p* < 0.0001), while for RSV B ATCC, it increased from 860 to 2606 (*p* < 0.0001). Similarly, for the seasonal strains, RSV A exhibited an increase from 1855 to 3856 (*p* = 0.07), and RSV B increased from 430 to 1121 (*p* = 0.0132). Immunity against circulating RSV A prior to vaccination was notably higher compared to other variants, whereas the baseline neutralizing capacity against seasonal RSV B was lower.

### 3.4. Impact of Clinical Factors on Antibody Response

**Figure 3:**
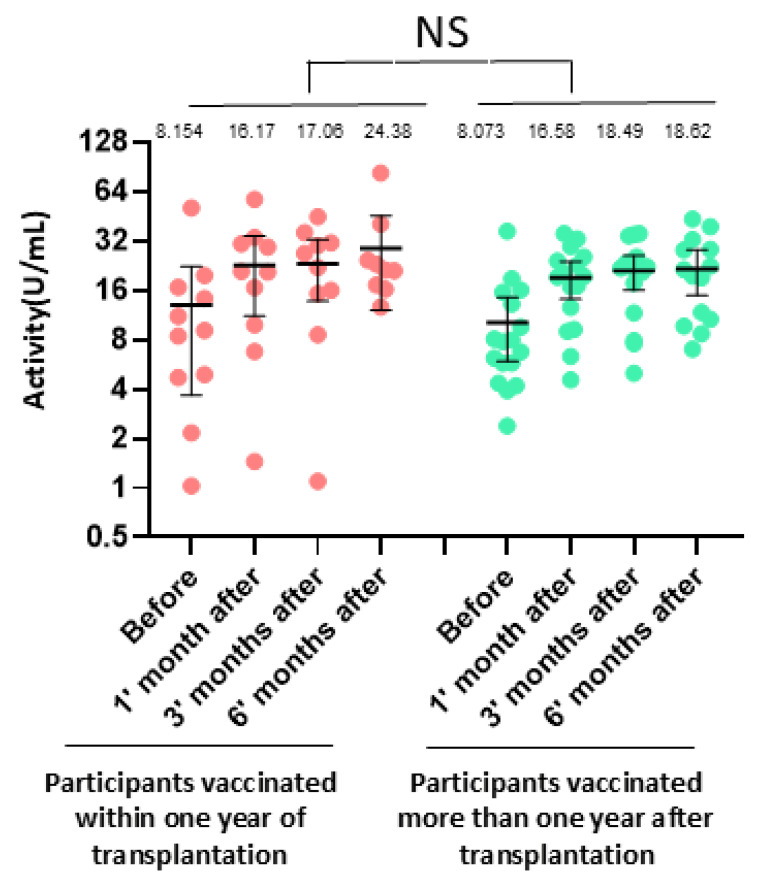
Antibody activity measured by ELISA in lung transplant recipients vaccinated within one year post-transplantation compared to those vaccinated more than one year post-transplantation. No statistically significant difference (NS) was observed between the groups (*p* = 0.3).

We compared antibody activity (ELISA) between participants vaccinated within one year of transplantation and those vaccinated more than one year after transplantation. No statistically significant differences were observed between the two groups (*p* = 0.3, *t*-test; Figure 3). Similarly, no significant differences in antibody responses were found between participants aged below and above 60 years.

### 3.5. Vaccine Tolerability and Reactogenicity

Pain was the most frequently reported injection-site reaction within 7 days after the vaccine dose, affecting eight patients (28.6%). Of these patients, four (14.3%) also reported injection-site redness or swelling.

## 4. Discussion

RSV poses a significant risk of LRTI, with considerable morbidity and mortality among LTx recipients [21]. In this study, we demonstrated a significant increase in antibody levels against RSV exceeding a predefined threshold, despite the immunosuppressed condition of the recipients. By six months, 74% of participants were seropositive for RSV compared to 24% at baseline. Importantly, we observed a marked rise in neutralizing antibodies, the key component of immune defense, while confirming the vaccine’s safety. Mild, localized side effects were the only adverse events reported in this previously unassessed population.

The RSV fusion (F) glycoprotein of the RSV-A strain (RSV-A A2 strain, ATCC No. VR-1540) was specifically chosen as the core component of the vaccine (RSVPreF3 antigen). This glycoprotein was selected due to its essential role in RSV entry into host cells and its high degree of conservation across RSV-A and RSV-B subtypes, making it an optimal antigen for broad protective efficacy [22,23,24,25,26]. RSV A and B ATCC strains are widely recognized as gold-standard reference strains for RSV assays. To evaluate vaccine efficacy, micro-neutralization assays were performed to measure the ability of serum antibodies to neutralize RSV virions and inhibit their entry and replication in host cells. These assays utilized historical RSV-A (Long, ATCC No. VR-26) and historical RSV-B (18537, ATCC No. VR-1580) strains and seasonal RSV A (R17535/Israel) and seasonal B (R15474/Israel) strains, demonstrating a significant post-vaccination increase in neutralizing capacity against both strains. Notably, the RSV-A VR-26 strain was selected for its genetic similarity to the vaccine strain.

Vaccination was associated with an increase in RSV-specific antibody levels against both RSV A and B ATCC strains, with protective effects persisting for at least six months. This aligns with the expectations, given that the vaccine contains a protein derived from the RSV-A ATCC strain. Beyond the ATCC strains, vaccination also resulted in an increase in neutralization efficiency against genetically diverse circulating seasonal RSV strains. Neutralizing capacity against seasonal RSV A prior to vaccination was more pronounced compared to other variants. This may explain why the increase in neutralizing titers against this strain six months post-vaccination, while above a two-fold change, was not statistically significant. In contrast, pre-vaccination immunogenicity against the seasonal RSV B strain was less pronounced compared to both the reference strains and seasonal RSV A. Overall, neutralizing antibody titers against the four strains remained at least twofold greater than baseline levels throughout the six-month follow-up period. This suggests that the genetic differences between the ATCC reference strains and circulating variants do not significantly impair antigen–antibody interactions. These findings highlight the vaccine’s ability to generate robust immune responses against both reference and seasonal RSV strains, suggesting a potential role in immune priming. Further studies are needed to evaluate the long-term durability of this cross-protective immune response and its implications for broader population immunity.

Adjuvant vaccines, which incorporate agents to amplify the immune response, are recognized as effective in immunocompromised patients, including those with substantial immune suppression, such as LTx recipients [27,28]. These vaccines enhance immunogenicity by overcoming the diminished immune-responsiveness characteristic of this population, often resulting in higher and more durable antibody levels. The immunogenicity of non-adjuvant RSV vaccines in immunocompromised individuals remains largely unexplored. In the absence of an adjuvant, these vaccines may elicit suboptimal antibody responses or reduce the durability of immunity, raising concerns about their efficacy in high-risk groups. Comparative clinical studies are necessary to evaluate whether non-adjuvant formulations can provide equivalent immunogenicity to adjuvant vaccines, particularly in the context of vulnerable populations where RSV poses significant morbidity and mortality risks. Such data will help clinicians make informed decisions about optimal vaccination strategies for immunocompromised patients.

The GSK study recruited healthy individuals aged 60 years or older and measured neutralizing antibody titers at baseline and one month post-vaccination, reporting significant increases against RSV A and RSV B (10.2-fold and 8.6-fold, respectively) [17]. In contrast, our study, which followed LTx recipients for six months post-vaccination, showed a milder but significant enhancement in neutralization capacity at one month post-vaccination (3.7-fold for RSV A and 3.5-fold for RSV B). By three months post-vaccination, antibody levels further increased (6.5-fold for RSV A and 6.9-fold for RSV B), suggesting a sufficient and prolonged immune response to the vaccine—an important observation not addressed in the GSK study. This relatively attenuated rise in neutralizing antibodies in our cohort compared to the healthy participants in the GSK study is likely attributable to the effects of immunosuppressive therapy.

No statistically significant differences were observed in RSV antibody levels between patients vaccinated within the first year post-transplant and those vaccinated more than a year after transplantation. This finding is noteworthy, as the first year post-transplant is typically characterized by the administration of the highest doses of immunosuppressive medications, which could theoretically impair the production of vaccine-induced antibodies. This lack of a significant difference suggests that the immune response to vaccination may not be as heavily influenced by the intensity of immunosuppressive therapy as previously anticipated. However, this observation should be interpreted cautiously, as the small sample size in this study may have limited the statistical power to detect subtle differences between these groups. Larger studies are needed to confirm these findings and better understand the impact of immunosuppressive regimens on vaccine efficacy in LTx recipients, particularly during the critical early post-transplant period.

This study has several limitations that should be considered. The relatively small sample size limited our ability to evaluate specific subgroups, such as older individuals and those stratified by time since transplantation. Additionally, the vaccine was administered in February 2024, near the end of the RSV season in Israel, which restricted our ability to assess its impact on reducing LRTI or acute RSV-related illness in this population. Because no RSV cases were recorded during follow-up, vaccine efficacy against clinical endpoints such as RSV-related LRTI or hospitalization could not be evaluated.

Neutralizing antibody titers, while commonly used as a surrogate marker of vaccine-induced immunity, may not fully capture real-world protection, and comparisons across studies should be interpreted with caution due to inter-laboratory variability in virus neutralization test (VNT) methodology. Furthermore, the use of ATCC strains—although widely used and clinically relevant—differs from those employed in some vaccine trials (e.g., the GSK study), which may affect cross-study comparisons.

In addition, the study did not include a control group of immunocompetent individuals, limiting our ability to compare the magnitude and kinetics of vaccine-induced responses between transplant recipients and the general population. Lastly, we did not assess cellular immune responses or antibody subclass distributions, both of which are critical components of antiviral defense and may contribute to the overall effectiveness of vaccination, particularly in immunocompromised hosts.

Despite these limitations, this study provides valuable preliminary insights into the safety, tolerability, and immunogenicity of the Arexvy vaccine in lung transplant recipients—a population at high risk for RSV-related complications and previously excluded from clinical trials.

In summary, this study demonstrated high seropositivity rates and significant increases in neutralizing antibody levels at 1, 3, and 6 months post-vaccination in LTx recipients. Antibody responses were sustained throughout the six-month follow-up, indicating durable immunogenicity. Importantly, no significant safety concerns were identified, supporting the vaccine’s tolerability and potential utility in this immunocompromised population.

## Figures and Tables

**Table 1:** Baseline patient characteristics.

	N = 28
**Baseline clinical characteristics**	
Recipient age at transplant, years, median (IQR)	59 (50–65)
Recipient age at time of vaccination, years, median (IQR)	62 (53–67)
Time from transplant to vaccination, days, median (IQR)	486 (243–966)
Sex, male, n (%)	21 (75)
Lung transplant type	
Bilateral lung transplant	25 (89.3)
Single lung transplant	3 (10.7)
Native lung disease, n (%)	
Pulmonary fibrosis	19 (67.9)
Chronic obstructive pulmonary disease	4 (14.3)
Cystic fibrosis	2 (7.1)
Other	3 (10.7)
CMV serology, n (%)	
D+R−	2 (7.1)
D+R+	20 (71.4)
D−R+	6 (21.4)
D−R−	0
Injection-site reaction, n (%)	
Pain	8 (28.6)
Swelling or erythema	4 (14.2)

CMV = cytomegalovirus; IQR = interquartile range; N = number.

## Data Availability

The data presented in this study are available in this article.
